# In Vitro Assessment of Antifungal Caspofungin on *Leishmania donovani* Culture Isolation

**DOI:** 10.1155/2017/3873187

**Published:** 2017-12-17

**Authors:** Narayan Raj Bhattarai, Keshav Rai, Suman Rijal, Basudha Khanal

**Affiliations:** ^1^Department of Microbiology, B. P. Koirala Institute of Health Sciences, Dharan, Nepal; ^2^Department of Internal Medicine, B. P. Koirala Institute of Health Sciences, Dharan, Nepal

## Abstract

*Leishmania* parasite isolation from the human aspirates is always challenging due to most probability of the fungal contamination and the use of antifungal drug which could support the selective growth of the *Leishmania* parasite. In this study, we examine the effect of antifungal drug caspofungin on the promastigote stage of *Leishmania donovani*. Promastigote parasite was cultivated in M199 + 20% heat-inactivated fetal calf serum and plated in 96-well plates. Seven different concentrations of caspofungin (512 *µ*g/ml to 8 *µ*g/ml) were exposed to parasites, and 50% inhibitory concentration (IC_50_) was calculated. *Candida* spp. was used in the experiments to know the efficacy of caspofungin to inhibit fungal growth. The IC_50_ values of *Leishmania* strains ranged from 23.02 to 155.80 *µ*g/ml (mean 90.25 ± 39.01 *µ*g/ml), and it was significantly higher (*P* value = 0.02) than IC_50_ values of *Candida* spp. (ranged from 0.001 to 0.12 *µ*g/ml, mean = 0.05 ± 0.05 *µ*g/ml). The reduced growth rate of the parasite was found with exposure to 50 *µ*g/ml of caspofungin. Growth inhibition of *Leishmania donovani* is significantly lower with caspofungin and could be used to protect the parasite cultivation from fungal contamination.

## 1. Background

*Leishmania* parasite cultivation in routine diagnostic and research laboratories is facing a major problem of microbial contamination despite strict adherence on aseptic microbiological practices. In general, the common antibiotics like penicillin and streptomycin are frequently used to prevent the bacterial contamination, but in particular these drugs have also limited a spectrum of inhibition to rule out the possibility of contamination. In contrary, fungal contamination during the parasite cultivation is another challenging arm in most of the laboratories. Yeast and yeast-like organisms are always reported as a major source of contamination during parasite culture. In fact, the antifungal compounds are rarely used to prevent fungal contamination in parasite culture. Recently, the report of cell banks showed that 39% of specimens are contaminated over a 2-year period and fungi were identified in 8% of these [1]. Polyene macrolide drugs (amphotericin and nystatin) have been used to reduce fungal contamination in cell line cultures [2, 3], and they target ergosterol in the fungal cell membrane. Lipid composition in *Leishmania* parasite cell membrane is similar that in fungi and yeast [4]. These drugs have also been shown to interact with Toll-like receptors and CD14 to induce signal transduction and release of inflammatory cytokines [5]. Hence, these drugs significantly affect parasite's cell physiology that can disrupt parasite cell cycle. Knowing the fact of antifungal antibiotics that are also potential inhibitors to the parasite growth itself, the exploration of antifungal compound which is not inhibiting the parasite growth could prevent the fungal contamination.

Caspofungin, an echinocandin drug, is used clinically for the invasive fungal disease. It kills the fungi by inhibiting 1-3-*β*-D-glucan synthesis in fungal cell wall [6]. It has been reported that caspofungin can effectively inhibit several contaminating clinical isolates of *Apergillus* spp., *Penicillium*, etc. [7]. The mammalian cell lines have found high resistance towards caspofungin [8]. The cells of *Leishmania* parasite are more similar to the mammalian cells. Therefore, the current study has an aim to determine the effect of caspofungin on *Leishmania* parasite as a proof of principle for their application in the culture of *Leishmania* parasite in order to avoid fungal contamination.

## 2. Materials and Methods

### 2.1. Parasite Promastigote Cultivation

The clinical isolates of *L. donovani* were isolated from the bone marrow of visceral leishmaniasis patients using the Tobie's agar with Locke's overlay. Later on, the culture was transferred to M199 with 20% fetal calf serum and cryopreserved at −80°C with 10% glycerol. The parasite was taken out of cryobank and M199 (Sigma-Aldrich, Cat. No. 2520), supplemented with 20% heat-inactivated fetal calf serum (Invitrogen), and was used to maintain promastigote parasite. 5 × 10^5^ parasite per/ml was inoculated in final 5 ml medium and incubated at 26°C [9]. The growth curve of the parasite was maintained for a week to determine the parasite growth rate. The *Leishmania* species isolates were determined by using the PCR assays targeting *L. donovani*(*donovani*) specific ribosomal DNA and followed by the PCR-RFLP heat shock protein 70.

### 2.2. Fungi Cultivation

*Candida* spp. was isolated from the clinical specimen at BPKIHS by Sabouraud dextrose agar (SDA). *Candida* species was confirmed by using germ tube test, sugar fermentation, growth at 45°C on SDA broth, and assimilation tests with xylose and α-methyl-D-glucoside. It was transferred to M199 with 20% heat-inactivated fetal calf serum (invitrogen) with 5 × 10^5^ parasite per/ml and then incubated at 26°C [10]. The growth rate of *Candida* was determined by maintaining the growth curve for a week.

### 2.3. Preparation of Drug

50 mg of caspofungin (Merck, USA) was dissolved in 5 ml sterile distilled water (10 mg/ml) and mixed thoroughly for 5 minutes. The drug solution was sterilized through a filter of pore size 0.2 *µ*m (PVDF), and drug solution was preserved at −80°C.

### 2.4. Drug Inhibition Measurement

#### 2.4.1. Leishmania Parasite

Log-phase promastigote parasites from third day of subculture were centrifuged at 3200 rpm for 10 minutes; parasites were counted and diluted to make 10^6^ parasite/ml. 100 *µ*l of parasites was plated out in duplicate on sterile 96-well plates (Corning Costar, USA, Cat. No. CLS3596). Each well was topped with 100 *µ*l of seven different concentrations of caspofungin ranging from 512 *µ*g/ml to 8 *µ*g/ml, including one negative control. The 96-well plates were covered with a sealing tape and incubated at 26°C for 4 days. Finally, viable parasites after exposure to caspofungin were determined by the trypan blue assay.

#### 2.4.2. Candida spp

Day 2 subculture of *Candida* was centrifuged at 3200 rpm for 10 minutes and resuspended to final cell density 10^6^ cells/ml. 100 *µ*l of *Candida* was transferred to duplicate wells of 96-well plates. Each well was topped with 100 *µ*l of seven different concentrations of caspofungin ranging from 8 *µ*g/ml to 0.00051 *µ*g/ml. The 96-well plates were covered with a sealing tape and incubated at 26°C for 3 days. Then, viable fungal cells after exposure to caspofungin were determined by the trypan blue assay.

### 2.5. Trypan Blue Viable Count Assay

The viable cells were counted in each well using the trypan blue (0.2%) dye exclusion method, where viable parasite became colourless and dead cells appear blue [11].

### 2.6. Calculation of IC_50_

Viable count of each well was entered in Microsoft Excel 2007, and log transformation was made for each drug concentration. These values were transferred in a GraphPad Prism version 5, and IC_50_ values were analysed for each strain using sigmoidal dose-response model (nonlinear regression) for caspofungin assay [12].

## 3. Statistical Analysis

All data were subjected to statistical analysis using Graphpad Prism Version 5. *P* values were calculated by Student's *t*-test or analysis of variance depending on the data. *P* values of less than 0.05 were considered as significant.

## 4. Results

**Figure 1:**
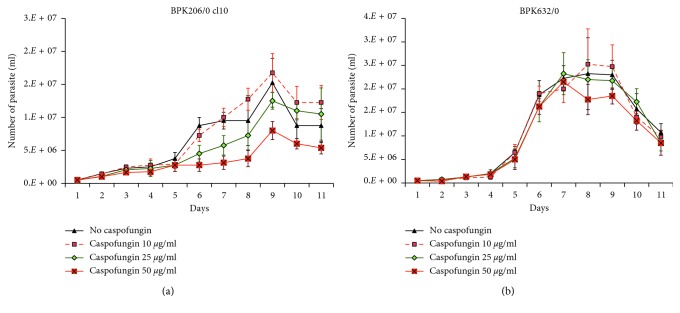
Growth curve of *Leishmania* parasite with different concentrations of caspofungin. Error bar indicates the 95% confidence interval of the mean based on triplicate culture of different strains. (a) BPK206/0 cl10 growth curve. (b) BPK632/0 growth curve.

The two *Leishmania* strains BPK206/0 cl10 and BPK632/0 were tested to determine IC_50_ against caspofungin, and detailed results were presented in Table 1. The IC_50_ values of *Leishmania* strains ranged from 23.02 to 155.80 *µ*g/ml (mean = 90.25 ± 39.01), as shown in Table 1. The parasite growth curve was also maintained with different concentrations of caspofungin (10 *µ*g/ml, 25 *µ*g/ml, and 50 *µ*g/ml) along with control. Caspofungin concentration more than 25 *µ*g/ml significantly inhibits the parasite growth than others, as shown in Figure 1.

IC_50_ values of *Candida* spp. were ranged from 0.001 to 0.12 *µ*g/ml (Table 1). The IC_50_ of *Candida* is significantly lower than *Leishmania* strains (*P* value = 0.02).

## 5. Discussion

In vitro cultivation of slow-growing pathogenic protozoan parasites is a difficult process as compared to bacterial and fungal culture. In fact, the longer generation time and requirement of complex nutrients are the main reasons for slow growth in parasite culture. Hence, it requires to maintain an extensive aseptic procedure during the primary isolation and culture maintenance in order to avoid contamination. However, abundant numbers of viable organisms are essentially required not only for confirmatory diagnosis but also for several downstream applications and drug susceptibility assay. Among others, *L. donovani* was most commonly cultivated in modified Tobie's blood agar at different tropics, but it was most commonly observed with yeast-like contamination during the isolation and maintenance of the parasite. The contamination in parasite culture could be due to the humid environmental condition to support rapid growth of yeast in the different endemic zones. Moreover, the lack of well-equipped laboratory facility to process the clinical specimen for in vitro culture would be another factor for the contamination. Therefore, there is requirement of certain antifungal agents which have no inhibitory effect to the *L. donovani* culture and also protect from the fungal contamination. That is why we undertook to assess the inhibitory effect of antifungal drugs in *L. donovani* during the parasite cultivation.

Our experimental results showed that *L. donovani* had significantly higher IC_50_ to caspofungin than *Candida* spp. which indicates that the *Leishmania* parasite had higher tolerance properties to caspofungin. Both *Leishmania* and *Candida* cells are eukaryotic in nature, but the *Candida* cell contains the cell wall but *Leishmania* lacks the cell wall. In *Candida*, the enzyme 1-3-*β*-D-glucan synthase in cell membrane has essential roles in cell wall formation. Since the caspofungin has a potential power to inhibit the function of 1-3-*β*-D-glucan synthase, it inhibits the growth of *Candida*. Therefore, the least growth inhibition was found in *L. donovani* isolates due to the lack of 1-3-*β*-D-glucan synthase. In addition, the growth curve of *Leishmania* culture isolates was also assessed in the presence of three different caspofungin concentrations (10 *µ*g/ml, 25 *µ*g/ml, and 50 *µ*g/ml). It showed that 50 *µ*g/ml caspofungin could inhibit a few strains of *Leishmania*, and no inhibition was determined in less than 50 *µ*g/ml of caspofungin. In contrast, *Candida* growth was inhibited with lesser concentration of caspofungin, and no growth was observed in culture incorporated with 10 *µ*g/ml caspofungin. Moreover, *Leishmania* parasite growth can adapt in five times more concentration of caspofungin than the *Candida* spp. We found that it could be advantageous to *Leishmania* culture procedure in order to prevent fungal contamination since *Leishmania* parasite growth can tolerate 25 *µ*g/ml concentration of caspofungin. Hence, 25 *µ*g/ml caspofungin concentration could be useful to overcome the fungal contamination in *Leishmania* culture although further study with large sample is required.

*Leishmania* parasites have digenetic life stage, and they are transforming from nonmotile amastigote to motile promastigote stage during in vitro isolation of the parasite from clinical specimens [9]. Caspofungin must not inhibit the parasite promastigote transformation from the beginning of isolation attempts in order to use against the fungal contamination since the *Leishmania* promastigote transformation might be inhibited by a few antibiotics [13]. The efficacy on the development of *Leishmania* promastigote in the presence of caspofungin was also examined. But, there was no significant reduction in the development of promastigote in parasite culture from clinical specimen (data not shown). Furthermore, *Leishmania* cell morphology in viable cultures was examined by phase-contrast microscopy, and promastigote cell morphology was not significantly different with/without caspofungin. This evidence indicates that the *Leishmania* parasite promastigote development was not negatively affected by the caspofungin.

However, there could be a question of efficacy of the drug towards other fungal contaminations rather than *Candida*. Undoubtedly, it could also inhibit the growth of all fungi since the key enzyme 1-3-*β*-D-glucan synthase is essential for the cell wall formation in all fungus and yeast. The fungal growth inhibition of *Apergillus* spp. and *Penicillium* spp. [7] was already reported in other than *Candida* spp. In conclusion, in this study, we show that caspofungin could be the potential agent for the selective growth of *L. donovani* culture isolation since it has no inhibitory effect towards the concentration in which the fungal cell viability is terminated.

## Figures and Tables

**Table 1:** IC_50_ of strains tested with caspofungin.

Strain	IC_50_ *µ*g/ml (mean ± SD)	Range (*µ*g/ml)
BPK206/0 cl10	61.70 ± 35.97	23.02 to 121.90
BPK632/0	118.8 ± 3.25	46.87 to 155.80
*Candida* spp.	0.05 ± 0.07	0.001 to 0.12
